# Biosynthesis of 2-Heptanone, a Volatile Organic Compound with a Protective Role against Honey Bee Pathogens, by Hive Associated Bacteria

**DOI:** 10.3390/microorganisms9112218

**Published:** 2021-10-26

**Authors:** Maria Ludovica Saccà, Giulia Bianchi, Roberto Lo Scalzo

**Affiliations:** 1 Council for Agricultural Research and Economics (CREA), Research Center for Agriculture and Environment (AA), Via di Corticella 133, 40128 Bologna, Italy; 2 Council for Agricultural Research and Economics (CREA), Research Centre for Engineering and Agro-Food Processing (IT), Via G. Venezian 26, 20133 Milan, Italy; giulia.bianchi@crea.gov.it (G.B.); roberto.loscalzo@crea.gov.it (R.L.S.)

**Keywords:** natural compounds, chemical signal, microbial communication, biosynthetic potential, honey bee microbiota, *Varroa destructor*

## Abstract

Beehives are populated by bacterial species with a protective role against honey bee pathogens thanks to the production of bioactive metabolites. These compounds are largely unexploited despite their high potential interest for pest management. This study evaluated the capability of bacterial species associated with honey bees to produce 2-heptanone, a volatile organic compound with anesthetic properties of the parasitic mite *Varroa destructor*. The production of this compound was quantified by SPME-GC-MS in a culture filtrate of nine bacterial strains isolated from the surface of honey bees, and the biosynthetic potential was evaluated in bacterial species associated with apiaries by searching for protein homologs putatively involved in its biosynthesis by using biocomputational tools. The findings pointed out that 2-heptanone was produced by *Acetobacteraceae bacterium*, *Bacillus thuringiensis* and *Apilactobacillus kunkeei* isolates in concentrations between 1.5 and 2.6 ng/mL and that its production was strain-specific. Putative methylketone synthase homologs were found in *Bacillus*, *Gilliamella*, *Acetobacteraceae*, *Bartonella* and Lactobacillaceae, and the protein sequence results were distributed in nine Sequence Similarity Network (SSN) clusters. These preliminary results support the hypothesis that 2-heptanone may act as a mediator of microbial relationships in hives and provide contributions to assess the role and biosynthetic potential of 2-heptanone in apiaries.

## 1. Introduction

Honey bees (*Apis mellifera* L., Insecta: Hymenoptera) are managed insects of economic and environmental importance for their role in pollination and for hive products. Over the past few decades in Europe and North America, their health has been threatened by multiple issues, including diseases, parasites, pesticides and environmental and socioeconomic factors [1]. Pests and pathogens, among which the mite *Varroa destructor* is one of the main causes of colony loss [2], have been controlled in the past years with the use of synthetic chemicals, but nowadays, the search for alternative strategies is of extreme interest to reduce the side effects and the development of resistance [3]. Natural products synthetized by living organisms are valuable resources that can be sustainable alternatives to chemicals [4]. Hundreds of signaling chemicals used by plants, insects and microorganisms for transmitting information between individuals of the same species or of different species (semiochemicals) have been discovered, but their use in pest management is still largely unexploited, despite the high potential interest [5,6]. In the hive environment, chemical signals from multiple biological interactions are countless [6,7,8]. Hundreds of VOCs have been identified in hive products, but studies have focused mainly on their involvement in the aroma of honey and on their use to obtain information on the botanical and geographical origins of honey [9]. Few studies have investigated the functional role of specific volatile compounds in plant–pollinator–predator–microorganism interactions. In this study, we focused on a semiochemical of honey bees, 2-heptanone (heptan-2-one or methyl n-amyl ketone). This methylketone secreted by the mandibular glands of worker honey bees has a debated role; it is thought to signal aversive situations by inducing defensive responses [10], to act as a chemical marker on visited flowers [11] or even as an adaptative modulator of learning and memory [12]. It has been recently discovered that honey bees use their mandibles to bite and remove *Varroa* mites from the bodies of their nest mates and that the released 2-heptanone acts as a local anesthetic, causing paralysis and death of this parasite [13]. The use of 2-heptanone as a mite repellent has been proposed, taking into consideration that this compound would not be toxic towards bees or contaminate hive products, since it already exists in hives [14,15]. 

Methylketones can be synthetized by bacteria, fungi, plants, insects and mammals and have been found in numerous natural environments, having various biological roles [16]. In plants, for example, these compounds are highly effective for protection against pests [17]. 2-heptanone produced by lactic acid bacteria (i.e., *Lactobacillus casei* and *Lactobacillus paracasei*) is known to contribute to aroma development in many dairy products [18]. Furthermore, this volatile compound was found to be synthetized by species of *Bacillus* during host–pathogen interactions [16] and by *Bacillus amyloliquefaciens* with antifungal properties against *Fusarium oxysporum* [19]. Even though this volatile compound has various bioactive roles of potential practical interest, its biosynthetic pathway has been scarcely investigated. In 2005, two methylketone synthetases from wild tomatoes (*Lycopersicon hirsutum*) were reported to use intermediates of the fatty acids biosynthetic pathway to synthesize methylketones [17]. In bacteria, a methylketone synthase involved in the production of 2-heptanone was identified in *Bacillus nematocida* [16]. 

The mediation of extracellular bacterial metabolites in beneficial interactions with honey bees has been reported [20,21], but little is known on VOCs produced by bacterial populations inhabiting hives. The microbiota associated with honey bees has lately been the subject of an increasing number of studies, and the most frequently reported taxa include Lactobacillaceae, Bacillaceae, Acetobacteraceae, Bifidobacteriaceae, *Gilliamella* and *Fructobacillus* [22,23,24,25,26,27,28]. 

Hence, the aims of this study were to (i) establish and quantify the production of 2-heptanone in the culture filtrate by bacterial species isolated from honey bees and (ii) investigate the potential of honey bee-associated bacteria to produce this natural compound with a protective role against hive pathogens by using biocomputational tools.

## 2. Materials and Methods

### 2.1. Bacterial Cultures

Nine bacterial strains isolated from the surfaces of healthy honey bees in a previous study [27] were selected based on their abundance in the microbiota of bees for testing their capability to produce 2-heptanone in a liquid culture. The selected isolates from the CREA-AA bacterial collection were the following: two strains of *Acetobacteraceae bacterium* (BO_L(L)1 and IM_G(L)3); three strains of *Bacillus thuringiensis* (BI_G1, PD_L1 and RN_G(L)2); one of *Bifidobacterium asteroides* (LE_V(L)2) and three *Apilactobacillus kunkeei* (BO_G1, LE_L(L)2 and LG_V1). They were stored in FGYP broth [29] with 20% (*v*/*v*) glycerol at −80 °C, and prior to use, bacterial colonies were regenerated in the same medium containing 10 g of D-fructose, 10 g of D-glucose, 10 g of yeast extract, 5 g of polypeptone, 2 g of sodium acetate, 0.5 g of Tween 80, 0.2 g of MgSO_4_∗7H_2_O, 0.01 g of MnSO_4_∗4H_2_O, 0.01 g of FeSO_4_∗7H_2_O and 0.01 g of NaCl per liter, pH 6.8. This medium was the same selective medium used for isolating bacterial strains from honey bees; the use of fructose and glucose as growth substrates supported the optimal growth, considering that these isolates originated from a sugar-rich environment such as an apiary [27]. Liquid cultures for the analysis of 2-heptanone were obtained by growing bacterial isolates in FGYP broth (50 mL) at 30 °C under aerobic conditions and shaking at 125 rpm for three days to permit the fermentation of carbohydrates and accumulation of byproducts. After incubation, the concentration of the cells in the stationary phase was around 10^7^ cell/mL (OD 0.8). The cell-free supernatants (CFS) were recovered from the fermentation broth by centrifuging (14,000× *g*, 10 min), filter-sterilizing (using cellulose acetate syringe filters, 0.22-µm pore size, GVS Life Sciences, Bologna, Italy) and keeping them at −80 °C until analysis.

### 2.2. GC-MS Analysis of 2-Heptanone

The analysis was performed following the method of Reference [16], with modifications. Each sample was composed of 10 mL of CFS in a 20-mL glass vial, with 2 g of NaCl added and closed with an aluminum–silicone/PTFE septum. The extraction was carried out using a DVB/CAR/PDMS solid-phase micro extraction (SPME) fiber (Supelco, Milan, Italy) exposed to the sample headspace at 60 °C for 30 min. Volatile compound desorption was obtained by exposing the fiber in the GC injector at 200 °C for 5 min. GC-MS analyses were carried out with an Agilent 6890 N GC connected to an Agilent 5973 mass spectrometer (Agilent Technologies, Cernusco sul Naviglio, Italy) and equipped with a DB-1 column (60 m × 0.25 mm I.D., film thickness 0.25 µm) in splitless mode using He as the carrier gas (flow 1 mL/min). The column temperature program was: 40 °C for 5 min, 3 °C/min to 180 °C and 8 °C/min to 220 °C for 5 min. The injector and detector temperatures were 200 and 230 °C, respectively, interconnecting the line temperature at 200 °C. The MS settings were as follows: filament voltage, 70 eV; scan range, 39–450 amu and scan speed, 1.4 scan/s. Uninoculated growth medium was used as the control. 2-Heptanone was identified by comparing its mass spectra with that stored in the Wiley 7n library and analyzing the authentic standard. Quantification was performed by the interpolation of a calibration curve made with known concentrations of 2-heptanone (Sigma-Aldrich, Italy), and the values were expressed as ng/mL; the detection limit was below 0.2 ng/mL.

### 2.3. Sequence Analysis 

A protein sequence BLAST was performed against the nonredundant protein database using acyl-CoA thioester hydrolase QBO55937 as the input to search for potential homologs in bacterial species populating apiaries. The search was oriented towards species reported to be associated with honey bees [25,26,27]. The following organisms were included in the search: Acetobacteraceae (taxid:433), *Bacillus* (taxid:1386), *Bartonella* (taxid:773), Bifidobacteriaceae (taxid:31953), *Bombella* (taxid:1654741), *Fructobacillus* (taxid:559173), *Gilliamella* (taxid:1193503), *Lactobacillus* (taxid:1578), *Lactobacillus kunkeei* (taxid:148814), *Leuconostoc* (taxid:1243), *Parasaccharibacter* (taxid:1602345) and *Snodgrassella* (taxid:1193515). The sequence of a thioesterase-like protein reported to synthesize methylketone using intermediates of the fatty acids biosynthetic pathway from *L. hirsutum* was also included in the dataset. 

The GenBank protein accession IDs of 72 sequences, with a minimum 23.5% identity, 3×10^−8^ E-values and 52% query coverage, were then submitted to the Enzyme Similarity Tool for generating Sequence Similarity Networks (SSNs) for visualization of the relationships among the protein sequences by grouping together the most related proteins in the clusters [30]. An alignment score threshold of 35 and a minimum length of 100 and maximum length 280 were set up for the analysis, and the networks were visualized in Cytoscape (v3.80). The obtained SSN was then used as the input for generating the Genome Neighborhood Diagrams (GNDs).

The evolutionary distances of the amino acid sequences were computed in MEGA7 [31] using the Poisson correction model with 1000 bootstrap replications [32]. 

## 3. Results and Discussion

### 3.1. 2-Heptanone Production in Bacterial Cultures

**Figure 1:**
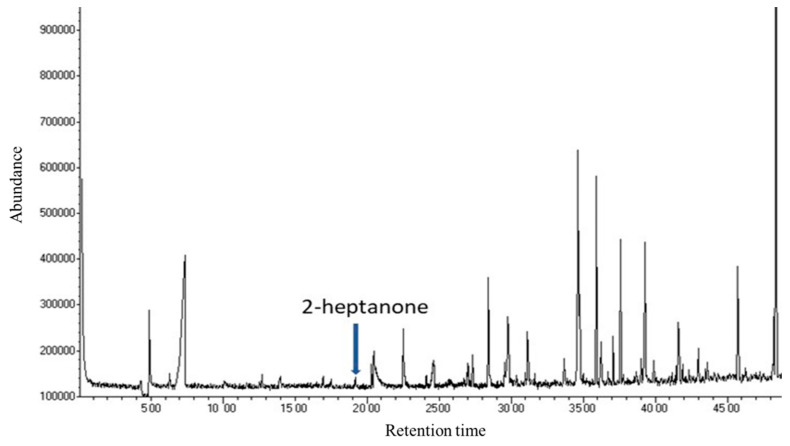
GC-MS chromatogram of the *Acetobacteraceae bacterium* strain IM_G(L)3 culture filtrate.

This study aimed at the identification and quantification, in bacterial culture filtrates, of 2-heptanone, whose protective role against honey bee pathogens has been previously reported [13]. The GC-MS analysis of CFS from the selected bacterial strains revealed a complex mixture of volatile compounds; among them, 2-heptanone was found at the retention time of 19.10 min (Figure 1). The gas chromatographic profile comprised many other compounds potentially interesting for their bioactivity; among which were 2,5-dimethylpyrazine at a retention time of 20.46 min, phenyl methanol (retention time of 26.90 min) and phenyl ethanol (retention time of 31.13 min). Interestingly, ant-associated bacteria have been reported to produce volatile pyrazines, including 2,5-dimethylpyrazine, previously identified as ant trail and alarm pheromones [33]. These compounds will be the object of further investigations on bioactive volatiles produced by hive-associated bacteria. Most of the other identified peaks are referred to as volatile fatty acids produced by microbial fermentation.

**Figure 2:**
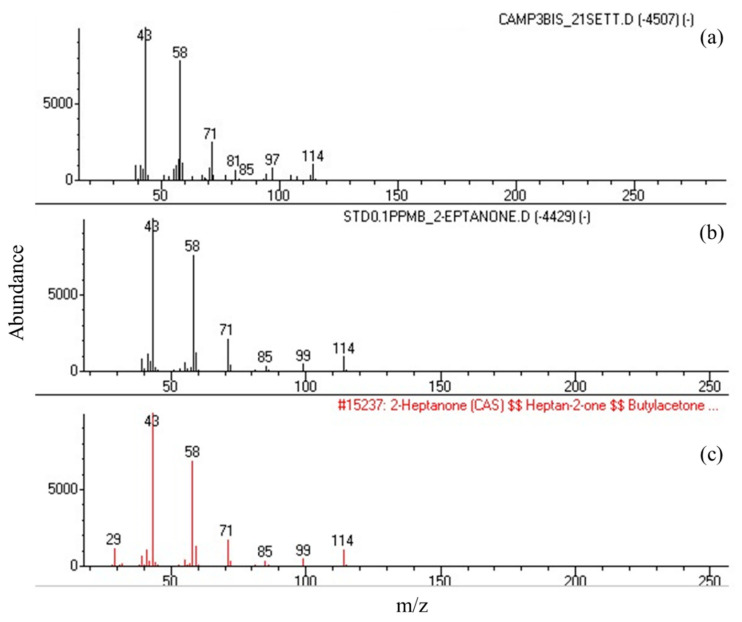
Comparison of the mass spectra of 2-heptanone from the SPME extract of the *Acetobacteraceae bacterium* strain IM_G(L)3 culture filtrate (**a**) and from the synthetic standard (**b**) to the spectrum stored in the Wiley 7n library (**c**). *m*/*z* = mass/charge.

The mass spectra of 2-heptanone from the extract of bacterial culture filtrates corresponded to that from the synthetic standard and to the spectrum in the Wiley 7n library, thus confirming the specificity of the results (Figure 2).

2-heptanone was found to be produced by both tested *A. bacterium* strains, by one *B. thuringiensis* and by one *A. kunkeei* in concentrations ranging from 1.5 to 2.6 ng/mL (Table 1). Traces were found in the culture filtrate of *B. asteroides* and of the other two *A. kunkeei* strains. The production of this compound therefore seemed to be strain-specific in the case of *B. thuringiensis* and *A. kunkeei*, since it was found only in one out of three isolates in both cases. 

Aliphatic ketones are historically known as insect alarm pheromones. Previous quantitative data, measured on insects, gave higher concentrations of these ketones than the present ones. In extracts of heads of *Atta texana* and *Atta cephalotes* ants, 2-heptanone was determined at around 160 ppb and in trace amounts, respectively [34]. In hives, the role of 2-heptanone appears to depend on its concentration; it is thought to be attractive in low concentrations and repulsive in higher concentrations, but contrasting results have been reported so far. It was originally hypothesized that this compound, secreted from the glands in honey bee mandibles, acted as an alarm pheromone stimulating defensive reactions against a potential threat [35,36]. Nevertheless, Papachristoforou and colleagues (2012) [13] demonstrated that it triggered no defensive responses when applied at colony entrances in doses of 0.001, 10 and 1000 µL, whilst it acted as a local anesthetic of Varroa mites when 0.061 µL of pure compound were applied topically on mites. In hive applications of up to 500 µL of pure 2-heptanone, it resulted that at no time did all bees fully or permanently exit the observation hive [14]. The low concentrations of 2-heptanone produced by the bacterial strains investigated in this study (<3 ng/mL), together with the high volatility of this compound, imply no side effects on the bees. Furthermore, in hives, its evaporation due to high temperature and humidity would be so quick that an investigation was specifically performed to maintain the compound longer to use it as a mite repellent [15]. The results of this study rather suggest that 2-heptanone may have a role in microbial chemical communication, and further studies are needed for the exploitation of 2-heptanone-producing bacteria for the control of honey bee pests and pathogens.

In this study, 2-heptanone production by *A.* *bacterium* was reported for the first time. This result is particularly interesting, since this species has been found to be associated with honey bees in different environments and suggested to have a beneficial role towards these insects [26]. Within Lactobacillaceae, 2-heptanone has been previously found to be produced by a honey bee bacterial symbiont *Apilactobacillus apinorum*, which showed antimicrobial properties against clinical isolates of pathogenic wound bacteria [37]; this is the first report of 2-heptanone production by *A. kunkeei*.

### 3.2. Biosynthetic Genes

**Figure 3:**
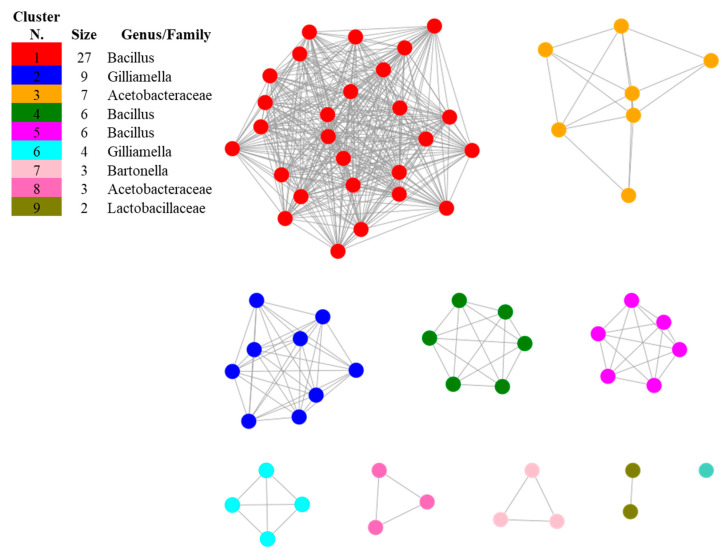
Sequence Similarity Network (SSN) showing the relationships among the protein sequences of putative homologs of methylketone synthase in bacterial species inhabiting apiaries. The most related proteins are grouped together in clusters. The singleton was constituted by the sequence of *Lycopersicon hirsutum* included in the dataset as the reference of a methylketone synthase described in plants.

The investigation of methylketone synthase proteins showed that putative homologs are encoded in the genomes of the bacterial taxa populating apiaries, such as Acetobacteraceae, *Bacillus*, *Bartonella*, *Bombella*, *Gilliamella* and *Parasaccharibacter* and that no significant similarities were found by restricting the search to *A. kunkeei*, *Bifidobacterium*, *Fructobacillus*, *Leuconostoc* and *Snodgrassella*. The presence of 2-heptanone in the culture filtrates of *A. kunkeei* and of *B. asteroides* in traces suggests that these species may use a different biosynthetic pathway to synthetize this compound yet to be described. After length filtering, 68 sequences were submitted to SSN analysis and resulted distributed in nine SSN clusters and one singleton (Figure 3 and Table 2). The only protein that did not cluster with the others was that of the wild tomato *L. hirsutum*, suggesting that putative methylketone synthase homologs evolved within the bacterial phylum, and a coevolutionary relation between honey bees and its associated microbiota may be hypothesized.

The first biggest cluster was composed by proteins from different species of *Bacillus*, including *B. nematocida* (Table 2), indicating that the proteins that are mostly related with putative methylketone synthase used as starting point in this study are found within this genus. The second cluster composed exclusively by *Gilliamella apicola* indicates that this species, widely distributed in the honey bee environment [23,24], is a potential producer of 2-heptanone. The compositions of the other minor clusters suggests that putative methylketone synthase homologs may be found in Acetobacteraceae, as well as in *Bartonella apis*, *Parasaccharibacter apium* and *Bombella apis*, well-known symbionts of honey bees [23,25,38,39], and, among Lactobacillaceae, in *Pediococcus acidilactici*, recently shown to have a protective effect towards honey bees [40].

Most of the bacterial thioesterases described in Table 2 have yet to be functionally characterized. The role of these enzymes has been studied more extensively in plants, where acyl thioesterases are reported to produce fatty acids that play roles in plant–insect and plant–microbial interactions [41]. A similar role of these enzymes in bacteria–bacteria and bacteria–insect interactions may be hypothesized and be the object of further studies.

**Figure 4:**
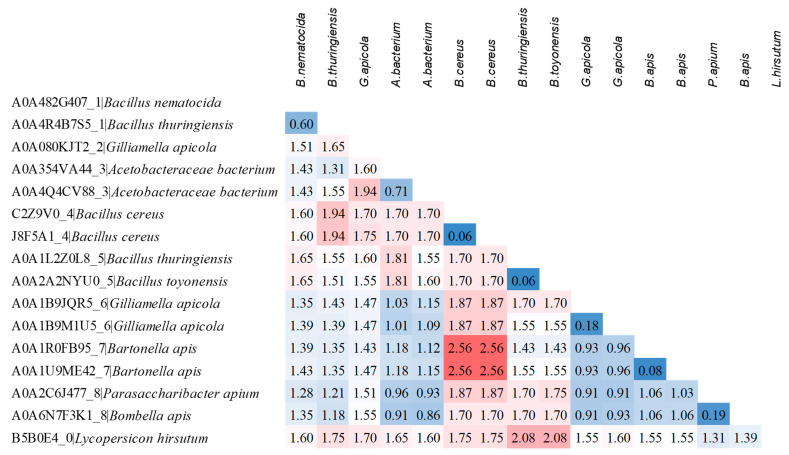
Estimates of the evolutionary divergences between a selection of representative protein sequences from each cluster. UniProt ID, cluster number and bacterial species are reported for each sequence. The number of amino acid substitutions per site from between sequences are shown. The values highlighted in blue indicate the most similar sequences, while those in red the most diverse.

The overall mean distance of the sequences listed in Table 2 was 1.33 ± 0.07, and the pairwise distance of the representative sequences from each cluster highlighted that the most diverse were those of *B. apis* from cluster 7 and *B. cereus* from cluster 4 (Figure 4).

**Figure 5:**
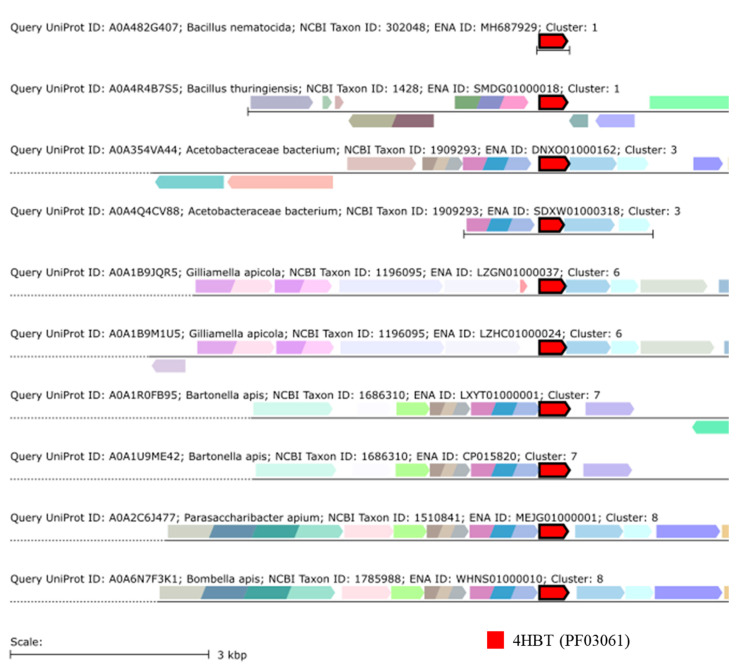
Genome Neighborhood Diagrams (GNDs) showing the 4HBT Thioesterase superfamily (PF03061) (red) in the genomes of the representative bacterial species from clusters 1, 3, 6, 7 and 8.

GNDs representing genomic regions around the genes encoded for the sequences from the submitted SSN highlighted the presence of two main protein families in the analyzed sequences: the 4HBT Thioesterase superfamily (PF03061), shared by clusters 1 (*Bacillus*), 3 (Acetobacteraceae), 6 (*Gilliamella*), 7 (*Bartonella*) and 8 (Acetobacteraceae) (Figure 5), and the 4HBT_2 Thioesterase-like superfamily (PF13279), shared by clusters 2 (*Gilliamella*), 4 (*Bacillus*) and 5 (*Bacillus*). The Acyl-ACP thioesterase (PF01643) protein family was found in Lactobacillaceae from cluster 9, suggesting that this family may have evolved separately. The fact that those thioesterase superfamilies were shared by different taxonomic groups, together with the high similarities of these sequences, reinforces the possibility of finding methylketone synthase homologs in the selected bacterial taxa. From a practical point of view, the presence of a conserved protein family putatively involved in the biosynthesis of methylketones in different bacterial taxa suggests the possibility of using this sequence fragment as a target when aiming at assessing the biosynthetic potential of 2-heptanone in apiaries. 

In a previous study, putative acyl-CoA thioesterase biosynthetic genes were sequenced from eight *B. thuringiensis* strains isolated from honey bees [42], among which were the three isolates tested in this study for 2-heptanone production. Therefore, the occurrence of this metabolite in the culture filtrate of only one out of three bacterial isolates suggests that the targeted acyl-CoA thioesterase genes may not always function as methylketone synthase and that the biosynthesis of 2-heptanone may be strain-specific and dependent from environmental factors. Certainly, studies oriented towards fermentation process optimization are needed for practical implications of 2-heptanone-producing strains in pest management in apiaries. 

Volatile compounds affecting the behavior of Varroa mites have been proposed as control strategies, but these semiochemical-based methods have received no validation in the field [8]. The use of 2-heptanone to control Varroa has been evaluated by the application of the pure compound in hives at mite-attracting and at miticidal concentrations [14], but issues have been encountered to ensure the persistence of this volatile compound at the desired levels. The possibility of using 2-heptanone-producing bacteria in naturally colonizing apiaries has so far never been evaluated. 

The production of 2-heptanone by two *A. bacterium* strains together with a high number of putative methylketone synthase homologs found within this species, as well as in Acetobacteraceae, suggests that active producers of this compound may be found within this family. Since the beneficial role of these bacteria towards honey bees has been frequently reported [26,43] but never associated with a particular mechanism, it may be hypothesized that this compound has a role in the interactions between Acetobacteraceae and honey bees. These results are in line with previous reports on the mediation of bee alarm pheromones in intra- and interspecies communications [7] and support the idea that microbially produced volatiles have an effect on honey bee physiology and behavior [44].

## 4. Conclusions

The production of 2-heptanone in culture filtrates of *A. bacterium*, *B. thuringiensis* and *A. kunkeei* isolates indicates that this bioactive metabolite is produced by honey bee-associated bacteria. Putative methylketone synthase homologs were found in bacterial taxa known to inhabit apiaries that were considered in this study, such as the *Bacillus* genus, Acetobacteraceae family and *Gilliamella* genus. The role of 2-heptanone in protecting honey bees from pathogens and as a chemical signal in microbial communication, as well as the optimization of the strain and fermentation process, and the biosynthetic pathway need further investigation. These preliminary results may find applications in the evaluation of the biosynthetic potential of the protective natural compound in apiaries.

## Figures and Tables

**Table 1:** Concentration of 2-heptanone in the bacterial culture filtrates (average of 2 replicates ± standard deviation). Tr = traces and n.d. = not detected.

Bacterial Strain	ng/mL
*Acetobacteraceae bacterium* BO_L(L)1	2.6 ± 0.7
*Acetobacteraceae bacterium* IM_G(L)3	1.9 ± 0.1
*Bacillus thuringiensis* BI_G1	n.d.
*Bacillus thuringiensis* PD_L1	2.2 ± 0.1
*Bacillus thuringiensis* RN_G(L)2	n.d.
*Bifidobacterium asteroides* LE_V(L)2	tr
*Apilactobacillus kunkeei* BO_G1	tr
*Apilactobacillus kunkeei* LE_L(L)2	tr
*Apilactobacillus kunkeei* LG_V1	1.5 ± 0.3

**Table 2:** List of potential methylketone homologs and their distribution in clusters, as calculated by the Sequence Similarity Network (SSN).

Cluster N.	UniProt ID	Description	Species
1	A0A084J093	Acyl-CoA thioester hydrolase| YbgC/YbaW family protein	*Bacillus mycoides*
1	A0A0B5WC13	Acyl-CoA thioesterase	*Bacillus thuringiensis*
1	A0A0D7XW04	Acyl-CoA thioester hydrolase	*Bacillus amyloliquefaciens*
1	A0A0G8F1L1	4-hydroxybenzoyl-CoA thioesterase	*Bacillus cereus*
1	A0A0J7ARV0	4HBT domain-containing protein	*Bacillus cereus*
1	A0A151V143	4-hydroxybenzoyl-CoA thioesterase family active site	*Bacillus cereus*
1	A0A162TGX7	Acyl-CoA thioesterase	*Bacillus cereus*
1	A0A1D3PLM8	Acyl-CoA thioesterase	*Bacillus toyonensis*
1	A0A1G4L174	YbgC/YbaW family acyl-CoA thioester hydrolase	*Bacillus cereus*
1	A0A1S7F927	4HBT domain-containing protein	*Bacillus thuringiensis*
1	A0A1T2PXZ8	Acyl-CoA thioesterase	*Bacillus cereus*
1	A0A1Y0XGB6	Acyl-CoA thioesterase YneP	*Bacillus amyloliquefaciens*
1	A0A242ZAB1	4HBT domain-containing protein	*Bacillus thuringiensis serovar* *londrina*
1	A0A243IF77	4HBT domain-containing protein	*Bacillus thuringiensis* subsp. *konkukian*
1	A0A243J5G2	4HBT domain-containing protein	*Bacillus thuringiensis* *serovar pirenaica*
1	A0A2A2P5K3	4HBT domain-containing protein	*Bacillus toyonensis*
1	A0A2I5JZ13	YbgC/FadM family acyl-CoA thioesterase	*Bacillus velezensis*
1	A0A2V1ZRD1	Acyl-CoA thioester hydrolase	*Bacillus* sp.
1	A0A482G407	Putative acyl-CoA thioesterase	*Bacillus nematocida*
1	A0A4R4B7S5	Acyl-CoA thioester hydrolase	*Bacillus thuringiensis*
1	A0A4Y6EXE5	Acyl-CoA thioesterase	*Bacillus tropicus*
1	A0A5B8PKI2	Acyl-CoA thioesterase	*Bacillus cereus*
1	A0A6D1TAX7	Acyl-CoA thioesterase	*Bacillus* sp.
1	A7Z572	YbgC/FadM family acyl-CoA thioesterase	*Bacillus velezensis*
1	B7IRQ3	Putative 4-hydroxybenzoyl-CoA thioesterase	*Bacillus cereus*
1	C3G682	4-hydroxybenzoyl-CoA thioesterase	*Bacillus thuringiensis* *serovar andalousiensis*
1	I2C620	4HBT domain-containing protein	*Bacillus amyloliquefaciens*
2	A0A080KJT2	Putative thioesterase	*Gilliamella apicola*
2	A0A1B9JPC2	4-hydroxybenzoyl-CoA thioesterase	*Gilliamella apicola*
2	A0A1B9JYC2	4-hydroxybenzoyl-CoA thioesterase	*Gilliamella apicola*
2	A0A1B9L9A8	4-hydroxybenzoyl-CoA thioesterase	*Gilliamella apicola*
2	A0A1B9LT81	4-hydroxybenzoyl-CoA thioesterase	*Gilliamella apicola*
2	A0A1B9M7F3	4-hydroxybenzoyl-CoA thioesterase	*Gilliamella apicola*
2	A0A1B9MQ32	4-hydroxybenzoyl-CoA thioesterase	*Gilliamella apicola*
2	A0A1B9MSC5	4-hydroxybenzoyl-CoA thioesterase	*Gilliamella apicola*
2	A0A1B9NKB4	4-hydroxybenzoyl-CoA thioesterase	*Gilliamella apicola*
3	A0A1M6K1F7	Acyl-CoA thioester hydrolase	*Roseomonas rosea*
3	A0A1Q2YQW1	Acyl-CoA thioester hydrolase YbgC	*Roseomonas* sp.
3	A0A1V2H861	4-hydroxybenzoyl-CoA thioesterase	*Roseomonas deserti*
3	A0A354VA44	Tol-pal system-associated acyl-CoA thioesterase	*Acetobacteraceae bacterium*
3	A0A379N4G1	Acyl-CoA thioester hydrolase YbgC	*Roseomonas mucosa*
3	A0A4Q4CV88	YbgC/FadM family acyl-CoA thioesterase	*Acetobacteraceae bacterium*
3	D5RRA5	Putative tol-pal system-associated acyl-CoA thioesterase	*Roseomonas cervicalis*
4	C2Z9V0	4-hydroxybenzoyl-CoA thioesterase	*Bacillus cereus*
4	J8F5A1	YbgC/YbaW family acyl-CoA thioester hydrolase	*Bacillus cereus*
4	J8LJ53	YbgC/YbaW family acyl-CoA thioester hydrolase	*Bacillus cereus*
4	R8L9R0	YbgC/YbaW family acyl-CoA thioester hydrolase	*Bacillus cereus*
4	R8P530	YbgC/YbaW family acyl-CoA thioester hydrolase	*Bacillus cereus*
4	R8QEN4	YbgC/YbaW family acyl-CoA thioester hydrolase	*Bacillus cereus*
5	A0A1L2Z0L8	1|4-dihydroxy-2-naphthoyl-CoA hydrolase	*Bacillus thuringiensis* subsp. *israelensis*
5	A0A2A2NYU0	Thioesterase	*Bacillus toyonensis*
5	A0A2A7HHQ3	Acyl-CoA thioesterase	*Bacillus cereus*
5	A0A2A7W716	Thioesterase	*Bacillus wiedmannii*
5	A0A2B8HQR2	Acyl-CoA thioesterase	*Bacillus thuringiensis*
5	A0A4U2U5Z0	Acyl-CoA thioesterase	*Bacillus cereus*
6	A0A1B9JQR5	Tol-pal system-associated acyl-CoA thioesterase	*Gilliamella apicola*
6	A0A1B9M1U5	Tol-pal system-associated acyl-CoA thioesterase	*Gilliamella apicola*
6	A0A1B9MHG5	Tol-pal system-associated acyl-CoA thioesterase	*Gilliamella apicola*
6	A0A1B9NL25	Tol-pal system-associated acyl-CoA thioesterase	*Gilliamella apicola*
7	A0A1R0FB95	(3S)-malyl-CoA thioesterase	*Bartonella apis*
7	A0A1U9ME42	(3S)-malyl-CoA thioesterase	*Bartonella apis*
7	A0A1U9MKW6	(3S)-malyl-CoA thioesterase	*Bartonella apis*
8	A0A2C6J477	4-hydroxybenzoyl-CoA thioesterase	*Parasaccharibacter apium*
8	A0A6N7F3K1	YbgC/FadM family acyl-CoA thioesterase	*Bombella apis*
8	A0A7U7G6Z5	4-hydroxybenzoyl-CoA thioesterase family active site	*Parasaccharibacter apium*
9	A0A062X6R3	Acyl-CoA thioester hydrolase| YbgC/YbaW family	*Ligilactobacillus animalis*
9	R0ET36	Acyl-CoA thioester hydrolase| YbgC/YbaW family	*Pediococcus acidilactici*
S1	B5B0E4	Thioesterase-like protein	*Lycopersicon hirsutum f* *glabratum*
